# *Oxyspirura petrowi* Causing Ocular Parasitism in a Free-Ranging Common Buzzard (*Buteo buteo*) in Romania and a Review of the Potential Zoonotic Implications as Cutaneous Larval Migrans

**DOI:** 10.3390/ani15111606

**Published:** 2025-05-30

**Authors:** Călin Mircea Gherman, Angela Monica Ionică, Katarzyna Anna Hołówka, Vlad Dan Cotuțiu, Carla Andreea Culda, Georgiana Iulia Lupu, Andrei Daniel Mihalca

**Affiliations:** 1 Department of Parasitology and Parasitic Diseases, University of Agricultural Sciences and Veterinary Medicine of Cluj-Napoca, Calea Mănăștur 3-5, 400372 Cluj-Napoca, Romania; calin.gherman@usamvcluj.ro (C.M.G.);; 2 Clinical Hospital of Infectious Diseases of Cluj-Napoca, 23 Iuliu Moldovan, 400348 Cluj-Napoca, Romania

**Keywords:** common buzzard, *Oxyspirura petrowi*, eyeworm, Romania

## Abstract

Bird eyes are more neglected organs in studies targeting the structure of the helminth fauna, which are instead mainly focused on the digestive and respiratory systems. In an extended study aimed at identifying helminths in the common buzzard (*Buteo buteo*), the eyes of 92 birds were also examined. Morphological characteristics and molecular analyses were used to identify the collected specimens. Eyeworms were found in only one bird, a juvenile male (1/92; 1.08%). Two nematodes were collected, a female and a fourth-stage larva. This is the first report of *Oxyspirura petrowi* in the common buzzard. The results highlight the need for careful examination of raptors’ eyes as ocular parasitism can affect their vision.

## Simple Summary

Birds are equally affected by thelaziid eyeworms as mammals are. *Thelazia* species, common in birds, are mainly distributed in the Neotropics, while *Oxyspirura* spp. are spread worldwide. Raptors are among the most affected species by eyeworms. This study aimed to morphologically and molecularly characterize two eyeworm specimens collected from common buzzards (*Buteo buteo*) in Romania. One of the 92 examined birds revealed eye parasitism (1/92; 1.08%). Morphologically, collected specimens resembled *Oxyspirura petrowi* and sequencing showed a 98.36% similarity to the same species. To our knowledge, this is the first report of *O*. *petrowi* in the common buzzard.

## 1. Introduction

The common buzzard, *Buteo buteo*, is a diurnal bird of prey and partial migrant that is spread almost throughout Europe, up to central Asia, and is present on several islands in the eastern Atlantic [1]. The species’ world population is estimated at 2,170,000–3,690,000 individuals [2], while European pairs range between 882,000 and 1,230,000 pairs [3]. In Romania, the evaluated population is 20,000–50,000 pairs, with an unknown, albeit likely, increasing trend [2].

The species is a generalist predator with an opportunistic feeding strategy. Its diet includes mammals, birds, reptiles, amphibians, arthropods, and fish, with predominance of the order Rodentia [4,5,6]. This varied diet exposes birds to the risk of contamination with a wide variety of helminths belonging to different taxa, Platyhelminthes, Nematoda, or Acanthocephala, confirmed by numerous studies [7,8,9,10]. These helminths most frequently affect the digestive system, with the respiratory system being less often parasitized. Other organs (kidneys, eyes, and brain) represent rare locations of metazoan parasites.

The eye is among one of birds’ rarely affected organs. Still, bird eyeworms belonging to the family Thelaziidae are well documented. The family includes two subfamilies, Oxyspirurinae and Thelaziinae, both containing genera and species that are ocular parasites of birds. Species from the subgenera *Thelaziella* and *Oxyspirura* are reported in diurnal raptors [11,12] and other bird orders, while *Ceratospira* and *Hempelia* species affect Columbiformes and Passeriformes [13,14]. While members of the family Thelaziidae are notorious, from a public health perspective, for transmitting the oriental eyeworm (*Thelazia callipaeda*), species reported in birds have yet to infect human eyes.

Given this context, this study aimed to confirm ocular parasitism with nematodes in common buzzards (*B*. *buteo*) from Romania by morphological and molecular characterization of the collected specimens.

## 2. Materials and Methods

### 2.1. Sample Collection, Origin, and Necropsy

The collected birds’ eyes were examined as part of a more extensive study to determine the structure of the common buzzard’s helminth fauna. The birds originated from all over the country and were found either as roadkill or died from other causes in various rehabilitation centers located in southern (Bucharest), northeastern (Piatra Neamț), western (Oradea), or central Romania (Tg. Mureș).

After collection, the birds were sent to the Parasitic Diseases Department of the Faculty of Veterinary Medicine in Cluj-Napoca. Initially, they were frozen at −18 °C. Subsequently, all birds were postmortem examined, and the eyeball and the appendages in the orbital cavity were individually collected. Afterward, the eyes and annexes were subjected to a parasitological search by opening them, flushing them with saline solution into Petri dishes, and checking under a stereomicroscope (Olympus SZ, Olympus Corporation, Tokyo, Japan). All nematodes were collected, washed with saline, morphologically identified, and later preserved in 70% ethanol for molecular identification.

The age of the birds was determined using a specific guide. Four age groups were recognized: juvenile, 2nd year autumn/3rd year spring, 3rd year autumn/4th year spring, and adults [15]. The sex was identified by genitalia visualization during the necropsy. The frequency (F), prevalence (P), geographical, and sex distribution of the eyeworm infections were assessed.

### 2.2. Morphological Identification and Characterization of Parasites

Morphological identification was based on the keys described by Addison and Anderson [16] and Pence [17]. Measurements were performed using an Olympus BX61 microscope (Olympus Corporation, Tokyo, Japan), with its dedicated software (Cell F version 3.1). Due to the reduced number of nematode specimens collected, limited morphometry was attempted, mainly targeting buccal capsule dimensions, esophagus length, striation length, and anal pore and vulva distance from tail end.

### 2.3. Molecular Characterization of Parasites

DNA was isolated from two specimens using a DNeasy Blood and Tissue kit (QIAGEN, Heildberg, Germany), and a ~700 bp fragment of the *cytochrome c oxidase* subunit 1 (*cox*1) was amplified using universal primers [18] and bilaterally sequenced using an external service (Macrogen Europe, Amsterdam, The Netherlands). The attained consensus sequences were compared to each other and to other isolates from the GenBank^®^ database by means of basic local alignment search tool (BLAST) analysis (https://blast.ncbi.nlm.nih.gov/Blast.cgi?PROGRAM=blastn&PAGE_TYPE=BlastSearch&LINK_LOC=blasthome, accessed on 10 April 2025).

The phylogenetic analysis was conducted in MEGA X software (version 10.2.6) [19]. The evolutionary history was inferred by the maximum likelihood method, using the Tamura–Nei model [20]. A discrete Gamma distribution was used to model evolutionary rate differences among sites (+*G*, parameter = 0.3697).

## 3. Results

A total of 92 specimens of common buzzards were collected between 2017 and 2025, from all over Romania. In terms of age, the fewest birds examined were juveniles (3/92; 3.3%), whilst at the opposite pole the age group from the second autumn year/third spring year (57/92; 62.0%), with intermediate values recorded for the other age categories (Table 1). Regarding the sex of examined birds, 55 were males (55/92; 59.8%) and 37 females (37/92; 40.2%).

**Figure 1:**
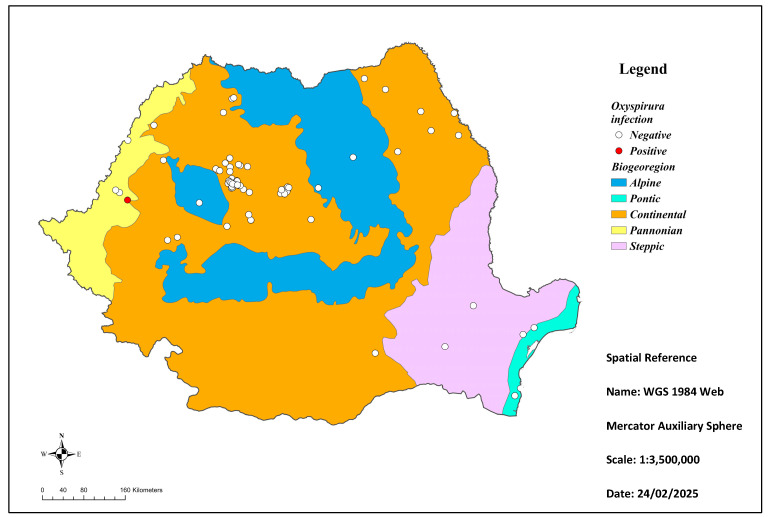
Ecoregional origin of the common buzzards collected from Romania.

Depending on the origin, 2 birds (2/92; 2.2%) were collected from the Alpine ecoregion and 82 from the Continental ecoregion (82/92; 89.1%), both situated in central Romania (Figure 1). Another three birds originated in Pannonian (west of the country) (3/92; 3.25%), two buzzards from Pontic (2/92; 2.2%), and the last three from the Steppic one (3/92; 3.25%), with the last two ecoregions both located in southeastern Romania. Ocular parasitism was revealed in only one specimen, meaning there was a prevalence of 1.08% (1/92). The bird was a juvenile male from the Pannonian ecoregion.

### 3.1. Morphological Description

**Figure 2:**
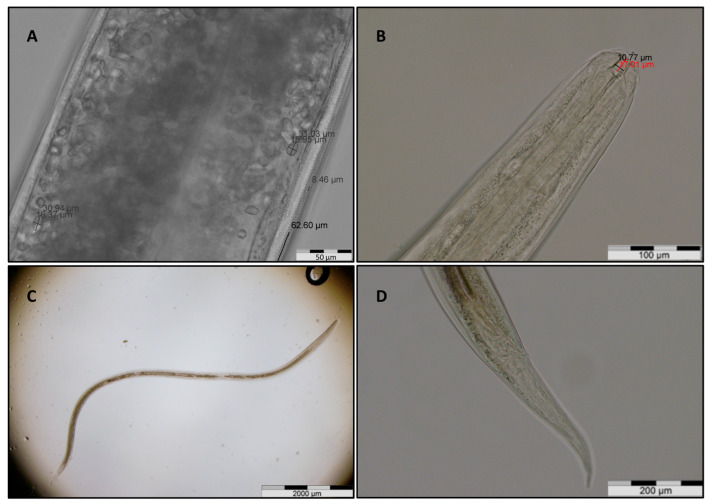
Morphological appearance and morphometric data of the identified eyeworms in a common buzzard from Romania. (**A**) Mid-section of the female specimen featuring eggs and striation measurements. B-D, buccal capsule and esophagus (**B**), overview (**C**), and tail end (**D**) of the L4 stage.

Two nematodes were recovered during sampling, one adult female and one L4 larva (Figure 2). While the L4 stage was intact, the female anterior end was sectioned off at the lower third of the esophagus. Therefore, the buccal capsule and some esophagus measurements could not be performed in the affected specimen (Table 2). Eggs within the female specimen’s uterus appeared unembryonated (Figure 2A).

### 3.2. Molecular Identification

**Figure 3:**
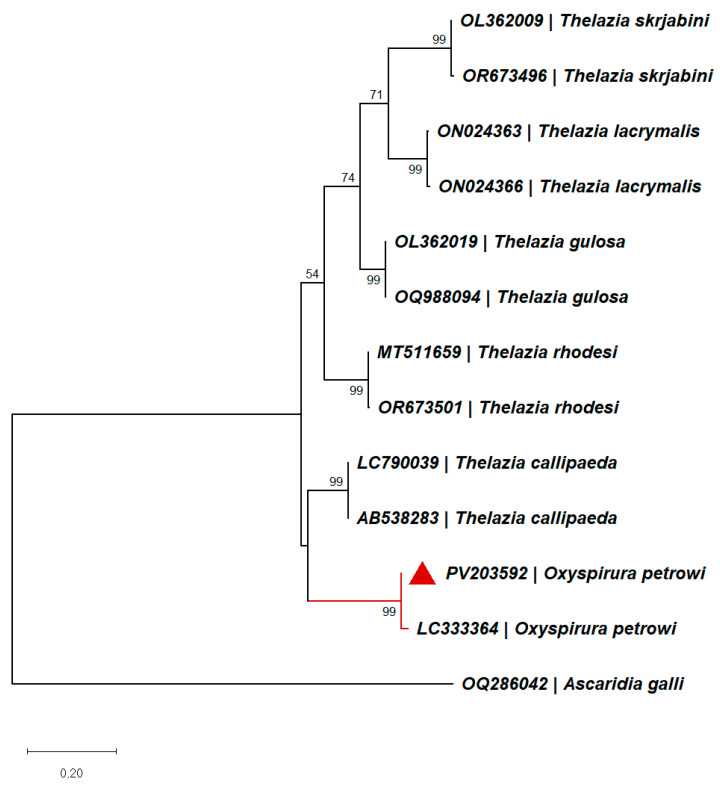
Bootstrap consensus tree inferred from 1000 replicates. The tree with the highest log likelihood is shown. The percentage of trees in which the associated taxa clustered together is shown next to the branches (values below 50 not shown). The tree is drawn to scale, with branch lengths measured in the number of substitutions per site. This analysis involved 13 nucleotide sequences. There were a total of 413 positions in the final dataset.

The two sequences were identical to each other and showed a 98.36% similarity to the only Oxyspirura petrowi cox1 sequence available in GenBank^®^ (Accession No. LC333364), with an overlap of 420/427 bp. The subsequent BLAST results include Spirocerca sp. (Accession No. KJ605486, KJ605489), with a similarity of 85.93% (586/661 bp overlap), followed by *Thelazia callipaeda* (Accession No. AM042550, AM042556, and MN719909), with a similarity of 85.17% (563/661 bp overlap). The currently attained sequence was deposited in GenBank^®^ under the Accession Number PV203592. Our sequence clustered together with the other *O. petrowi* isolate, forming a sister group of *T. callipaeda* (Figure 3).

## 4. Discussion

Eyeworm primarily refers to thelaziid nematodes that infect the orbital cavities, conjunctival sacs, and lacrimal ducts of mammals and birds, which are typical members of the Thelaziidae family. However, the eyes can also be affected by other parasites, such as *Philophthalmus* trematodes [21], tapeworm larvae [22], and spirurid nematodes like *Onchocerca lupi*, *Setaria* sp., and *Aprocta* sp. [23,24,25], or the protozoan *Toxoplasma gondii* [26]. Two genera of Thelaziidae, *Oxyspirura* and *Thelazia*, each divided into two subgenera, of which *Oxyspirura* and *Thelaziella* [27] are significant for bird eye parasitism.

Species of the subgenus *Thelaziella* Travassos, 1918 differ morphologically from *Thelazia* spp. through the presence of gubernaculum and differ ecologically due to their specificity for birds. Moreover, they have a restricted geographical distribution to the Neotropical realm, specifically South America, but have also reported in India [28], Japan [29], and Russia [30]. In South America, mainly in Brazil, 15 *Thelaziella* species were reported [11,14,31], but according to the newest nematode classification [27] only 7 species are recognized within the subgenus. However, the restricted distribution of *Thelaziella* species in birds, which is particularly limited to Brazil, is intriguing. Generally, thelaziid species are vectored by muscid and drosophilid flies [32,33,34] that take first-stage larvae and will later transmit infective third-stage larvae to a new host. In contrast, no information is available about the intermediate hosts of *Thelaziella* spp. It is possible that the restricted distribution can be explained by the intervention of intermediate hosts belonging to taxa similar to those of other thelaziids but having, in turn, a restricted geographical distribution. Another explanation could be the neglected nature of parasitological examination of the eyes in wild birds, thus underestimating eyeworm distribution.

In contrast, of the subgenus *Oxyspirura* Drasche in Stossich, 1897 contains 35 recognized species [27] with a cosmopolitan geographical spread. They parasitize a large variety of bird species belonging to approximately one-third of the bird orders, namely Accipitriformes [35], Bucerotiformes [35], Caprimulgiformes [31,36], Cariamiformes [37], Charadriiformes [35], Coraciiformes [38,39], Cuculiformes [31,40], Falconiformes [12,41], Galliformes [42,43], Gruiformes [40,44], Passeriformes [45], Pelecaniformes [40], Piciformes [40,46], Psittaciformes [47], Ralliformes [48], Strigiformes [49,50], and Trogoniformes [51]. This wide distribution and the polyspecific host–parasite relationships are favored by the biological cycle in which arthropods, predominantly from the Orthoptera and Blattodea orders, act as intermediate hosts [52]. These arthropods are part of the preferred regular diet of many bird species, including nocturnal and diurnal raptors [53]. The birds will be more easily contaminated by consuming arthropods compared to feeding-flies inoculation of *Thelaziella* larvae, especially when irritated birds become restless and flies’ feeding and larvae transfer can be consecutively interrupted. Two species of the subgenus are significant for birds’ parasitism, namely *O*. *mansoni*, found mainly in tropical and subtropical regions of America, Asia, and the Pacific Ocean, and *O*. *petrowi* predominantly spread in America, Europe, and Asia [54]. They are reported in various bird species, including multiple species of owls and other raptors [12,41,49].

Here, we report, to the best of our knowledge, for the first time, ocular parasitism with *O*. *petrowi* in the common buzzard. Still, apart from North America, the main geographic distribution of the species *O*. *petrowi* is reported in different passerines from Europe [55,56] and Asia [57,58]. Within the *Buteo* genus, there is only one report from Turkey, where *O*. *mansoni* was identified in a long-legged buzzard (*Buteo rufinus*) [12]. In the two specimens isolated in the current study, all measurements were consistent with findings from other recent studies [59], thus confirming the species is *O*. *petrowi*.

For animal parasitic nematodes, sequencing of mitochondrial genes such as *cytochrome c oxidase* subunit 1 (*cox*1) is widely used to determine lower taxonomic levels and resolve species-level phylogenies due to their high degree of variation [60]. A recent study evaluated six markers for representatives of three families of nematodes of veterinary and medical importance (30 species) and concluded that *cox*1 is a useful marker for diagnostic or metabarcoding purposes, firstly due to its high interspecific resolution and secondly due to the higher number of sequences available in databases [61]. However, in the present case, *cox*1 sequences for ocular parasites of birds in general were limited to a single entry of *O. petrowi*, isolated from a Northern Bobwhite quail, *Colinus virginianus*, in the USA [62]. The geographic distancing of the two specimens may account for the noted genetic divergence between them (seven SNPs, representing 1.64% of total overlap region). Due to the comparable variation in inter- and intra-specific distances among different lineages, there is no universal similarity ‘cut-off’ characteristic for the interspecific level [63]. Instead, several sister-species pairs would need to be used to determine the minimum level of genetic distance characteristic of species for that particular clade [64]. Nevertheless, in the present case, the species determination was based firstly on morphological determination, while the evolutionary analysis placed our specimen within the same clade as the American isolate.

In addition to affecting vision in parasitized vertebrate animals, the zoonotic potential of the species in the Thelaziidae family is significant. In this background, the transmission of the *Thelazia callipaeda* from wild and domestic canids, the primary definitive host, to humans by secretophagous flies is well known [65]. However, little is known about the zoonotic nature of the thelaziids parasitizing the birds. While there are no reports of *Thelaziella* species affecting people, human parasitism has recently been reported for the *Oxyspirura* genus in Vietnam [66]. Specifically, molecularly identified *O*. *petrowi* larvae caused a systemic cutaneous larval nematodiasis, clinically characterized by disseminated pruritic erythema, in a 41-year-old man from Son La Province, northern Vietnam. Since the patient’s neighbors also had the same condition, it was suggested that *Oxyspirura* sp. larval infection could be a public health concern. The lack of *Thelaziella* spp. reports in humans is strange since these species are more easily transmitted by flies compared with the orthopterans–oral transmission of *O*. *petrowi*. Food habits vary worldwide, including, in some areas, the consumption of arthropods [67]: the Vietnamese patient admitted to having eaten grasshoppers and crickets. Consecutively to this report, larval *Oxyspirura* spp. infection has been included in the possible etiology of cutaneous larval migrans (CLM) [68]. However, it cannot definitively include bird eyeworms in the list of potentially zoonotic nematodes. Still, a more precise diagnosis of CLM will be required, especially among farm workers in whom such cases were previously attributed to hookworms [69]. Furthermore, a more thorough molecular description of the genus *Oxyspirura* could provide much needed clarity into its phylogenetics.

## 5. Conclusions

This study reported eyeworm infection with *O*. *petrowi* for the first time in the common buzzard and for the first time in Romania. One adult female and a fourth-stage larva were collected from 1 juvenile male out of 92 examined birds, meaning a prevalence of 1.08%. Morphologically and molecularly, the species *O*. *petrowi* was confirmed. The results showed that this eyeworm can also affect raptors which are distant from a wide variety of other bird species. This emphasizes the need for careful eye examination in these birds to highlight such parasitism as the examination of the eyeball is often neglected.

## Figures and Tables

**Table 1:** Age, gender, and ecoregion distribution of the common buzzards collected from Romania.

Descriptor		Frequency (n/total)	Distribution (%)
age	Juvenile	3/92	3.3
	2nd-year autumn/3rd-year spring	57/92	62.0
	3rd-year autumn/4th-year spring	20/92	21.7
	Adults	12/92	13.0
sex	Males	55/92	59.8
	Females	37/92	40.2
ecoregion	Alpine	2/92	2.2
	Continental	82/92	89.1
	Pannonian	3/92	3.25
	Pontic	2/92	2.2
	Steppic	3/92	3.25

**Table 2:** Morphometry (in µm) of recovered *Oxyspirura petrowi* specimens.

Morphological Trait		Female	L4
Total body length		14,404	6010
Buccal capsule	Width	-	10.77
	Depth	-	17.01
Esophagus	Length	-	621.31
	Maximum width	101.45	60.7
Anal pore	Distance from tail end	290	284.87
Vulva	Distance from tail end	480	
Nerve ring	From anterior end of esophagus	-	140
Striations		6.2	4.5
Eggs	Length	30.94	-
	Width	16.37	-

## Data Availability

All data may be shared and should be requested from the corresponding author.
